# Synthesis, Characterization, and *In Vitro* Antimicrobial and Anticancer Evaluation of Copolyester Bearing 4-Arylidene Curcumin in the Main Chain

**DOI:** 10.1155/2014/495927

**Published:** 2014-10-29

**Authors:** Narendran Kandaswamy, Nanthini Raveendiran

**Affiliations:** Postgraduate and Research Department of Chemistry, Pachaiyappa's College, Chennai 600 030, India

## Abstract

Synthesis of random copolyester bearing 4-arylidene curcumin **M**_1_ in the polymer backbone was prepared by solution polycondensation method. The influence of copolyester bearing 4-arylidene curcumin **M**_1_ unit on the properties of copolyester such as inherent viscosity, solubility, and thermal stability was investigated and studied in detail. The inherent viscosity and polydispersity index of the copolyester were found to be 0.19 dL/g and 1.38, respectively. The chemical structure of the copolyester was investigated by Fourier-transform infrared spectroscopy (FTIR) and proton nuclear magnetic resonance (^1^H-NMR) spectroscopy. The physical properties of copolyester were characterized by thermogravimetric analysis (TGA), differential scanning calorimetry (DSC), gel permeation chromatography (GPC), and X-ray diffraction (XRD) technique. Agar disc diffusion method was employed to study the antimicrobial activity of the random copolyester. *In vitro* anticancer activity against lung cancer (*Hep-2*) cell line was investigated.

## 1. Introduction

Biocompatibility is an important aspect to be considered for an implantable biomaterial. The implanted biomaterial into a body initiates a host response that reflects the first steps of tissue repair. Generally after implantation of biomaterial, numerous types of host responses lead to the development of adverse effects such as blood-material interactions, acute inflammation, chronic inflammation, granulation tissue development, foreign body reaction [1], and biofilm formation due to microbes. Sometimes prolonged inflammation may lead to tumor in the surrounding tissue. Hence there is a requirement for an improved drug delivery device that is capable of delivering a drug having antimicrobial, anti-inflammatory, and antitumor effects in the vicinity of a surgical implant over a prolonged period or the usage of coated medical devices. Where in the coating includes the subject polymer matrix and a low solubility prodrug. Such coatings can be applied to surgical implements such as screws, plates, prosthesis, anchors, washers, sutures, staples, valves, and membranes. By considering all these facts, it is required to develop a polymeric biomaterial having very good antimicrobial and anticancer activity along with; it has to maintain its physical property such as good thermal stability, mechanical strength, solubility, and easy fabrication process. A probe through the literature indicates that there has been a rising interest in synthesizing aliphatic aromatic copolyester [2–5] as biomaterials in various medical disciplines. The incorporation of bulks [6, 7] such as 4-arylidene curcumin containing polar carbonyl groups along with introduction of flexible spacer [6, 8, 9] in the polymer chain tends to reduce the interaction between polymer chains and eventually leads to an increase in free volume and solubility with improvement in processability [10–13] by maintaining its thermal stability. Curcumin and its derivatives are one of the important classes of organic compounds possessing intense biological activity including antimicrobial, antibacterial, antifungal, anti-inflammatory, antitumor, and anti-HIV. Of particular interest, 4-arylidene curcumin possesses better bioavailability, without increasing toxicity.

The aim of the present investigation is to synthesise and characterize random copolyester containing biologically active 4-arylidene curcumin by inherent viscosity measurements, solubility tests, FTIR, ^1^H-NMR spectroscopy, X-ray diffraction analysis (XRD), thermogravimetric analysis (TGA), differential scanning calorimetry (DSC), and gel permeation chromatography. The synthesized copolyester was subjected to* in vitro* anticancer activity against lung cancer (*Hep-2*) cell line and antimicrobial studies.

## 2. Experimental

### 2.1. Materials and Methods

Curcumin (Aldrich) was used as such. p-Tolualdehyde, sebacoyl chloride, 1,6-hexanediol, DMAP, triethylamine, DMF, and piperidine (Aldrich) were used as received. Solvents were purified according to the standard procedure in the literature [14].* Hep-2 *cell lines were obtained from the National Centre for Cell Sciences, Pune (NCCS). The cells were maintained in minimal essential medium (MEM) supplemented with 10% FBS, streptomycin (100 *μ*g/mL), and penicillin (100 *μ*g/mL) in a humidified atmosphere of 50 *μ*g/mL CO_2_ at 37°C. MEM was purchased from Hi Media Laboratories. Fetal bovine serum (FBS) was purchased from Cistron Laboratories. Trypsin, methylthiazolyl diphenyl-tetrazolium bromide (MTT), and dimethyl sulfoxide were purchased from Sisco Research Laboratory Chemicals, Mumbai. Inherent viscosity was determined at a polymer concentration of 0.5 g/dL in DMF at 30°C using an Ubbelohde suspended level viscometer. 3% (w/v) solutions were taken as a standard for solubility of copolyester in various solvents. FTIR spectra were recorded on Perkin Elmer 883 spectrophotometer. ^1^H-NMR spectra for monomer and polymer were recorded on a Bruker 400 MHz spectrometer using CDCl_3_ and DMSO as a solvent. Thermogravimetric analysis (TGA) was performed in nitrogen at 10°C min^−1^ with a TGA instrument SDT Q600. Differential scanning calorimetry (DSC) was performed with DSC 200 F3 Maia instrument at a heating rate of 10°C min^−1^ under nitrogen atmosphere. Molecular weight of copolyester was measured on Waters 501 gel permeation chromatography equipped with polystyrene-divinylbenzene column and a differential refractive index detector. Polystyrene was employed as calibration standard and chloroform as solvent. X-ray diffraction measurements were recorded on a Bruker XRD D8 FOCUS using Cu K*α* radiation source. Antibacterial activity of random copolyester was determined by disc diffusion method on Muller Hinton agar (MHA) medium and Sabouraud dextrose agar (SDA) medium, respectively. Both Muller Hinton agar and Sabouraud dextrose agar media were poured into the Petri plate. After the medium was solidified, the inoculums were spread on the solid plates with sterile swab moistened with the bacterial suspension. The discs were placed in MHA and SDA plates and 20 *μ*L of sample concentration was added. Each sample was placed in the disc. The plates were incubated for 24 h, at 35°C. Then the antimicrobial activity was determined by measuring the diameter of zone of inhibition. Standard antibiotic ampicillin (20 *μ*g/disc) was used as reference. Fresh bacterial cultures of Gram positive bacteria* Bacillus subtilis *and* Staphylococcus aureus *and Gram negative bacteria, namely,* P. vulgaris *and* P. aeruginosa*, were used for the antibacterial test. Antifungal activity of extracts was determined by disc diffusion method on Sabouraud dextrose agar medium. Disc diffusion assay (DDA) can also be performed for screening by standard method. Stock cultures were maintained at 4°C on nutrient agar slant. Active cultures for experiments were prepared by transferring a loop full of culture from the stock cultures into the test tubes containing Sabouraud dextrose broth, which were incubated for 24 h at 35°C.* C. albicans *were the fungi which were used for the study.

The MTT assay was performed as first described by Mosmann with the modifications suggested by Denizot and Lang. Cells (1 × 10^5^/well) were plated in 24-well plates and incubated at 37°C with 5% CO_2_ condition. After the cell reaches the confluence, the various concentrations of copolyester** P**_1_ were added and incubated for 24 hours. After incubation, the sample was removed from the well and washed with phosphate-buffered saline (pH 7.4) or MEM without serum. 100 *μ*L/well (5 mg/mL) of 0.5% 3-(4,5-dimethyl-2-thiazolyl)-2,5-diphenyl-tetrazolium bromide (MTT) was added and incubated for 4 hours. After incubation, 1 mL of DMSO was added in all the wells. The absorbance at 570 nm was measured with UV-spectrophotometer using DMSO as the blank. Measurements were performed and the concentration required for a 50% inhibition (IC_50_) was determined graphically. The % cell viability was calculated using the following formula:

(1) $$\begin{array}{c} \%\;\text{cell}\;\text{viability}=\left(\frac{A_{570}\;\text{of}\;\text{treated}\;\text{cells}}{A_{570}\;\text{of}\;\text{control}\;\text{cells}}\right)\times 100. \end{array}$$

Graphs were plotted using the % of cell viability in *y*-axis and concentration of the sample in *x*-axis. Cell control and sample control were included in each assay to compare the full cell viability in cytotoxicity and anticancer activity assessments.

### 2.2. Synthesis of 1,7-Bis-(4-hydroxy-3-methoxy-phenyl)-4-(4-methyl-benzylidene)-hepta-1,6-diene-3,5-dione **M_1_**

In a three-neck round bottom flask containing curcumin (1 mmol, 3 g) in a mixture of chloroform/methanol (25 mL). A methanolic solution of p-methylbenzaldehyde (1 mmol, 0.978 g) was added with stirring. A small amount of catalyst (piperidine, 5% v/v) was added to the mixture which was then stirred at room temperature for further 48 h. The progress of the reaction was monitored by TLC [DCM-MeOH (99 : 1, v/v)]. After completion of the reaction, the product was precipitated by adding the reaction mass into a solution of 40 mL of hexane-chloroform (50 : 50, v/v) mixture, which resulted in** M**_1_, 1,7-bis-(4-hydroxy-3-methoxy-phenyl)-4-(4-methyl-benzylidene)-hepta-1,6-diene-3,5-dione. The product was separated and washed with hexane to get a pure product (yield 3.3 g, 86.8%). IR (KBr) *ν* cm^−1^ was 3456, 3078, 2898, 2363, 1657, 1486, 1356, and 1146.

^1^H NMR (400 MHz, DMSO-d6) *δ* was 2.35 (s, 3H), 3.85 (s, 6H), 6.88 (d, 2H), 6.90 (m, 4H), 7.2 (d, 2H), 7.55 (m, 2H), 7.88 (m, 4H), and 8.01 (s, 1H).

### 2.3. Synthesis of Copolyester (P_1_)

**Figure 1:**
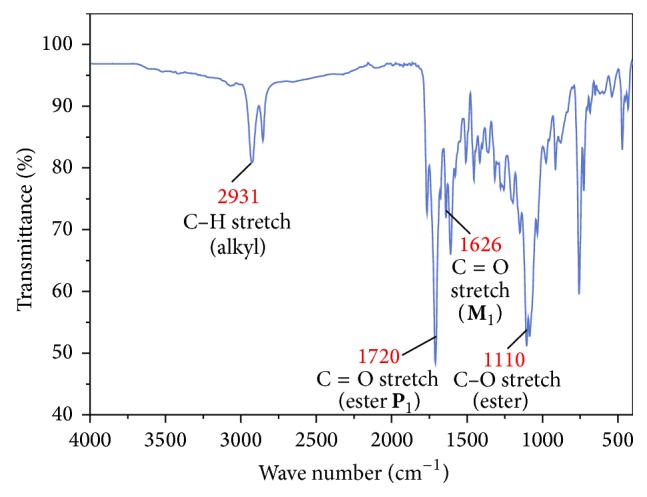
FTIR spectra of copolyester** P**_1_.

**Figure 2:**
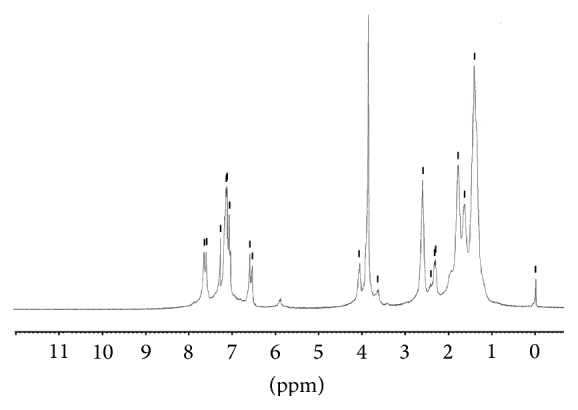
^1^H-NMR spectrum of copolyester** P**_1_ in CDCl_3_.

A mixture of 4-arylidene curcumin** M**_1_ (3 g, 1 mmol) and hexane diol** M**_3_ (0.75 g, 1 mmol) in 30 mL of DMF was taken in a three-neck round bottom flask. Temperature of the reaction mass was reduced to 15°C and charged with triethylamine (1.28 g, 2 mmol) and (0.5% w/w) DMAP. To this sebacoyl chloride** M**_2_ (3 g, 2 mmol) dissolved in dry DMF (15 mL) was added for 15 min with constant stirring for 24 h. The resulting reaction mass was filtered and the filtrate was poured into water (250 mL) to precipitate the random copolyester. It was then washed and dried under vacuum to get the product. The yield was almost quantitative. Spectral data are presented in Figures 1 and 2.

## 3. Results and Discussion

### 3.1. FTIR and ^1^H-NMR Analysis

**Scheme 1:**
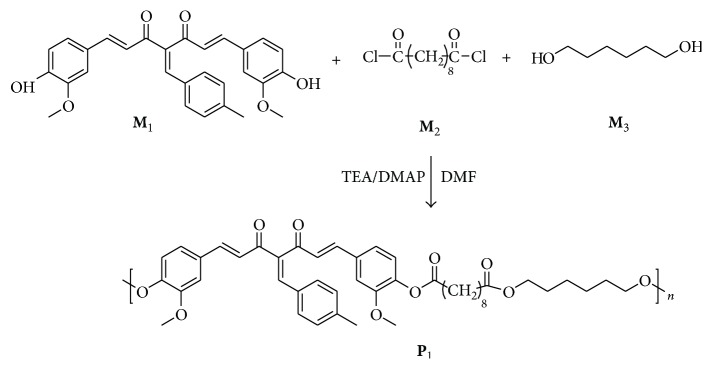
Synthesis of copolyester bearing 4-arylidene curcumin.

Structural assignments of the peaks coincide with the spectral data of the FTIR analysis shown in Figure 1. The copolyester shows characteristic absorption due to carbonyl stretching of the ester group at around 1720 cm^−1^. However, it is interesting to find the peak at 1626 cm^−1^ corresponding to the stretching of carbonyl group of *α*, *β*-unsaturated compound** M**_1_. So it can be inferred that the 4-arylidene curcumin molecule is an integral part of the polymer backbone. Stretching vibration of ester C–O appears at 1080–1110 cm^−1^. The copolyester** P**_1_ exhibits absorption band around 2850–2931 cm^−1^; this is attributed to the presence of –C–H stretching vibration of alkyl group in the polymer chain (see Scheme 1). No significant absorption in the OH stretching region was detected, confirming that the dihydroxy monomer** M**_1_ effectively got involved in polymerisation.

In Figure 2, ^1^H-NMR spectrum of the copolyester** P**_1_ was discussed. The characteristic peaks in the range of 7.04–7.80 ppm are due to the aromatic protons of monomer** M**_1_. The characteristic peak of curcumin at 9.70 ppm corresponding to the hydroxyl protons completely disappears after the reaction. The methoxy protons (–OCH_3_) of the monomer** M**_1_ group appeared at 3.91 ppm, respectively. The peak characterising the methylene groups of sebacoyl unit** M**_2_ and hexamethylene unit** M**_3_ at 1.22–2.58 ppm and the pendant methyl unit from monomer** M**_1_ got merged with the peaks of aliphatic methylene groups at the region mentioned above. It is explicit that the monomer** M**_1_ successfully incorporated into the polymer chains.

### 3.2. Polymer Solubility and Inherent Viscosity

Solubility parameter is an important criterion for polymer processing. The copolyester showed good solubility in chlorinated solvents and polar aprotic solvents; this may be due to the presence of pendant arylidene group in the monomer** M**_1_ which leads to reduced rigidity of polymer chains and hinders close chain packing, thereby reducing chain interactions. Thus it is inferred that the incorporation of arylidene pendant unit influence more in solubility. The inherent viscosity of the copolyester was determined in DMF at 30°C at the concentration of 0.5 g/dL using an Ubbelohde suspended level viscometer. The inherent viscosity of the copolyester was found to be 0.19 dL/g. In general inherent viscosities increase with increase in molecular weight. It is evident that copolyester exhibits lower viscosity value because the presence of unsymmetrical bulky monomer** M**_1_with arylidene pendant unit may tend to reduce the polymer chain length and hence inherent viscosity value decreases. It is also reflected in the GPC data. The number average molecular weight (M_*n*_) and weight average molecular weight (M_*w*_) of the copolyester** P**_1_ are 4,429 and 6,127 with 1.38 as polydispersity index value, respectively. It is inferred that the copolyester formed in a random manner and lower molecular weight of the polymer is suitable for the biological application.

### 3.3. DSC and TGA Studies

**Figure 3:**
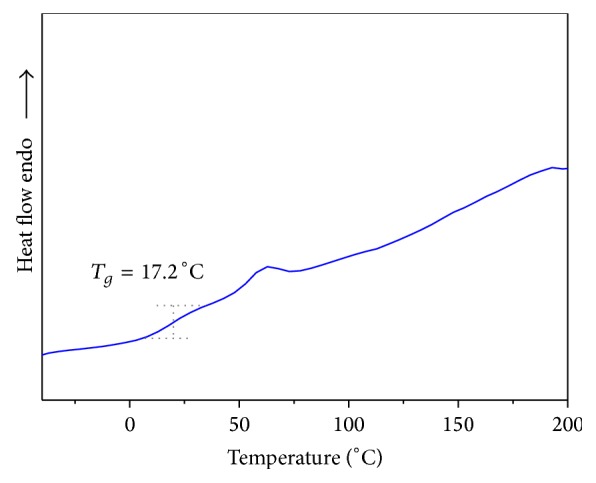
DSC thermogram of random copolyester** P**_1_.

**Figure 4:**
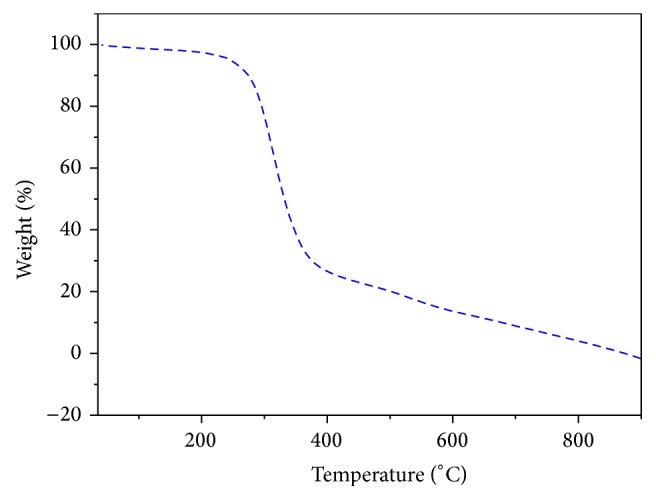
TGA curve of copolyester** P**_1_.

The influence of polymer structure on the thermal properties of copolyester** P**_1_ was investigated by thermogravimetric analysis (TGA) at a heating rate of 10°C min^−1^ in nitrogen atmosphere and the TGA curve is shown in Figure 4. The initial decomposition temperature (IDT), temperature for 10% weight loss (*T*_10_), and the maximum decomposition temperature (*T*_max⁡_) of copolyester are 225°C, 240°C, and 320°C, which indicates its good thermal stability. The *T*_*g*_ of the copolyester was determined by DSC at a heating rate of 10°C min^−1^ under nitrogen atmosphere and the DSC thermogram of copolyester** P**_1_ shows low glass transition temperature (*T*_*g*_) at 17.2°C and melting temperature (*T*_*m*_) at 67.5°C as shown in Figure 3. As expected the lowest *T*_*g*_ value was observed for copolyester, which can be attributed to the fact that the pendant arylidene unit from curcumin monomer** M**_1_ in the polymeric structure was unable to array regular and hinders the close chain packing, thus reducing the rigidity of the polymeric structure.

### 3.4. XRD

**Figure 5:**
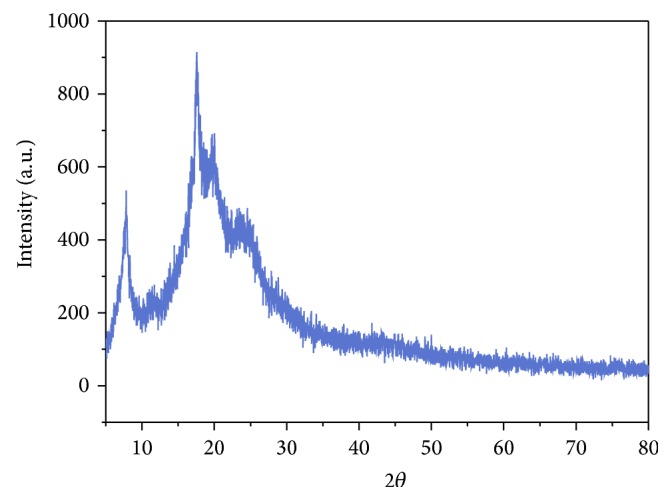
X-ray diffractogram of copolyester** P**_1_.

X-ray diffraction pattern of copolyester** P**_1_ exhibits the essential amorphous and semicrystalline nature. Apart from this, copolyester exhibited some degrees of crystallinity at 2*θ* range of about 5°, 17°, and 19°. This observation could be attributed to the introduction of methylene unit [6] from both acid dichloride** M**_2_ and diol** M**_3_ as shown in X-ray diffraction pattern. In addition, the presence of pendant arylidene unit from monomer** M**_1_ reduces coplanarity and hindered the dense packing of polymer chains and destroyed the ordered arrangement, resulting in amorphous nature of these polyesters, which is also reflected in their improved solubility. Copolyester** P**_1_ exhibits conspicuous amorphous halo at 2*θ* range of about 15° to 30° and all these facts are evident from X-ray diffractogram of** P**_1_ as shown in Figure 5.

### 3.5. Antimicrobial Activity

**Figure 6:**
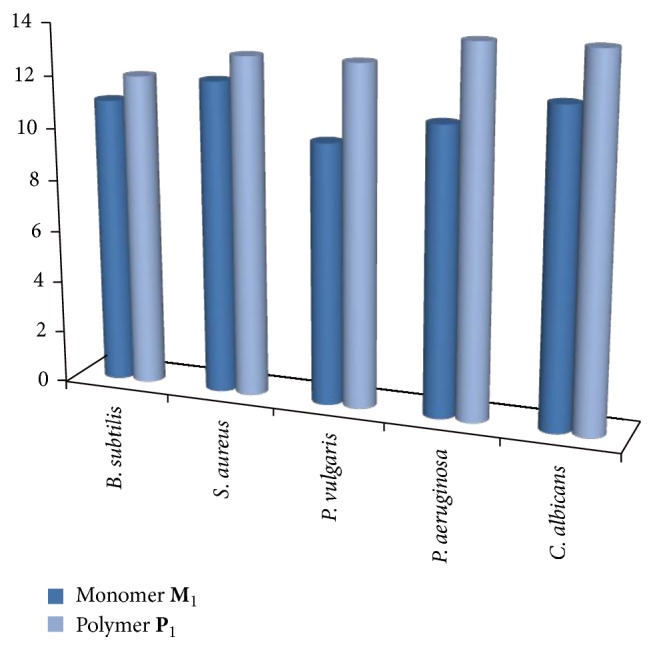
Comparative activities of monomer** M**_1_ and copolyester** P**_1_ against various microbes.

The results of antimicrobial activity are presented in Table 1 and the comparative activities of monomer** M**_1_ and copolyester** P**_1_ against microbes are shown in Figure 6. The monomer and the copolyester are active against all the microbes such as* B. subtilis, S. aureus, P. vulgaris, P. aeruginosa*, and* C. albicans *with inhibitory zone ranges of 9.0–13.0 mm and 9.0-14.0 mm, respectively. All the copolyesters exhibited enhanced antimicrobial activity compared with the monomer and this is due to the lipophilicity of the copolyester. In general more lipophilic compounds can penetrate greater the lipophilic cell membranes of Gram positive bacteria, while less lipophilic compounds are more liable to penetrate the cell wall of Gram negative bacteria. It is explicit from Figure 6. Copolyester** P**_1_ exhibits extreme antimicrobial activity and this is attributed to the ability of *α*, *β*-unsaturated ketone from curcumin to undergo a conjugated addition to a nucleophilic group like thiol group in an essential protein of the microorganism. The presence of more lipophilic methoxy moiety at the position 4,4′ of curcumin and small percentage of end phenolic and hydroxyl group of coumarin has contributed to the wide spectrum of antibacterial activities, thus proving their efficiency as antimicrobial polymers.

### 3.6. Anticancer Evaluation of Copolyester P_1_

**Figure 7:**
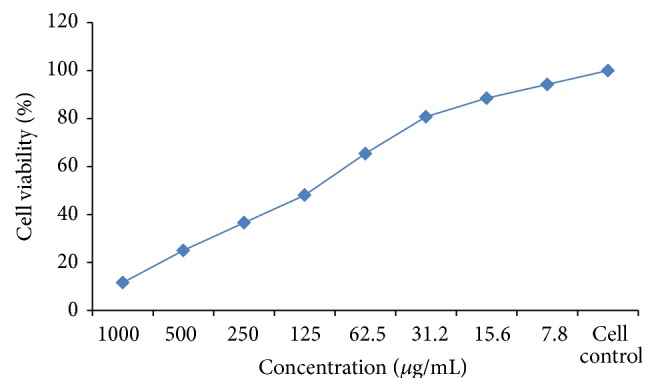
Graphical representation of activities of copolyester** P**_1_ in the MTT assay.

Viable cells were determined by the absorbance. Measurements were performed and the concentration required for a 50% inhibition of viability (IC_50_) was determined graphically. The effect of the copolyester on* Hep-2* cell line was expressed as the % cell viability. IC_50_ of the polymer was determined and was shown in Table 2. The assessment of cell viability by MTT assay through graphical representation is shown in Figure 7. The covalent conjugation of 4-arylidene curcumin through ester linkage increases its molecular size and steric hindrance may improve its cellular permeability and restrict its cancer progression [15, 16]. It is explicit from Table 2 that low concentration of copolyester** P**_1_ induced greater anticarcinogenic activity effects on* Hep*-*2* cell line than the individual agents. Literature survey reveals that the substitution of electron donating groups at the para position of benzene ring increases the antitumor and antioxidant activity of curcumin [17].

## 4. Conclusion

In summary, aliphatic aromatic random copolyester bearing 4-arylidene curcumin has been synthesised via solution polycondensation method. The copolyester showed good solubility in various organic solvents and this is attributed to the incorporation of pendant arylidene unit. The structure of the random copolyester was confirmed by FTIR and ^1^H-NMR spectroscopy. XRD pattern of the copolyester revealed that the copolyester was found to be amorphous with some degrees of crystallinity in nature. The enhanced antimicrobial and anticancer activity of the copolyester revealed that it can find versatile applications in various biomedical disciplines. Of particular interest, it can act as prodrug for the delivery of arylidene curcumin or prodrug carriers for other therapeutic agents.

## Supplementary Material

Supplementary data (FTIR spectra) of the monomer 1,7-Bis-(4-hydroxy-3-methoxy-phenyl)-4-(4-methyl-benzylidene)-hepta-1,6-diene-3,5-dione, M1 associated with this article can be found to be significant in discussing the structure of the copolyester.

## Figures and Tables

**Table 1:** Zones of inhibition (in mm) of the compounds against various microbes.

Compound	*B. subtilis *			*S. aureus *			*P. vulgaris *			*P. aeruginosa *			*C. albicans *		
	20 *μ*L	40 *μ*L	60 *μ*L	20 *μ*L	40 *μ*L	60 *μ*L	20 *μ*L	40 *μ*L	60 *μ*L	20 *μ*L	40 *μ*L	60 *μ*L	20 *μ*L	40 *μ*L	60 *μ*L
**M**_1_	9	10	11	9	10	12	9	9	10	10	10	11	9	9	12
**P**_1_	10	11	12	9	11	13	11	13	13	12	14	14	11	13	14

**Table 2:** Anticancer activities of copolyester **P**_1_ on *Hep*-*2* cell line.

S. number	Concentration (*μ*g/mL)	Dilution	Absorbance (O.D)	Cell viability (%)
1	1000	Neat	0.07	13.46
2	500	1 : 1	0.14	26.92
3	250	1 : 2	0.20	38.46
4	125	1 : 4	0.27	**51.92**
5	62.5	1 : 8	0.34	65.38
6	31.2	1 : 16	0.41	78.84
7	15.6	1 : 32	0.45	86.53
8	7.8	1 : 64	0.50	96.15
9	Cell control	—	0.52	100
