# Supplementary figures and images for: Synthesis, Characterization, and In Vitro Antimicrobial and Anticancer Evaluation of Copolyester Bearing 4-Arylidene Curcumin in the Main Chain
Source: Int Sch Res Notices. 2014 Oct 29;2014:495927. doi: 10.1155/2014/495927 (PMC4897485; doi:10.1155/2014/495927)

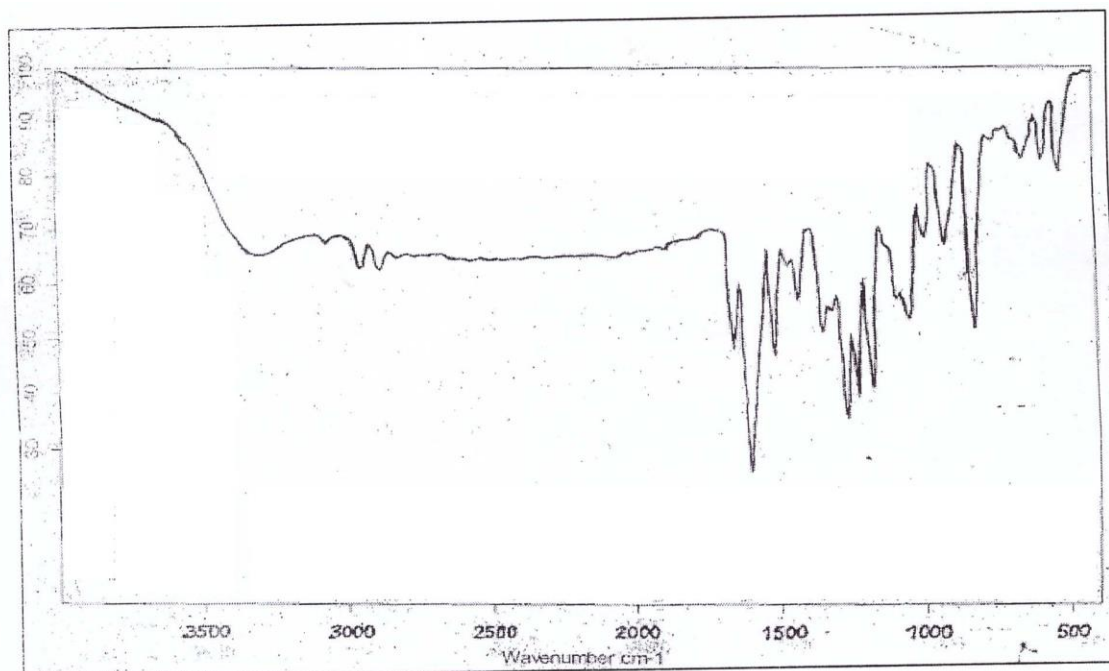

Supplement: Supplementary file 1 — Supplementary data (FTIR spectra) of the monomer 1,7-Bis-(4-hydroxy-3-methoxy-phenyl)-4-(4-methyl-benzylidene)-hepta-1,6-diene-3,5-dione, M1 associated with this article can be found to be significant in discussing the structure of the copolyester. [file 495927.f1.pdf]
